# Optical Bench Evaluation of a Novel, Hydrophobic, Acrylic, One-Piece, Polyfocal Intraocular Lens with a “Zig-Zag” L-Loop Haptic Design

**DOI:** 10.3390/vision8040066

**Published:** 2024-11-14

**Authors:** Andreas F. Borkenstein, Eva-Maria Borkenstein, Pooria Omidi, Achim Langenbucher

**Affiliations:** 1 Borkenstein & Borkenstein Private Practice, Privatklinik der Kreuzschwestern Graz, Kreuzgasse 35, 8010 Graz, Austria; 2 Department of Experimental Ophthalmology, Saarland University, 66424 Homburg, Germany

**Keywords:** presbyopia-correcting intraocular lens, polyfocal, optical bench, modulation transfer function, optical quality

## Abstract

Purpose: The number of presbyopia-correcting (premium) intraocular lenses (IOLs) is growing steadily as the desire for spectacle independence after cataract surgery increases. The aim of this laboratory study was to evaluate a newly launched hydrophobic, acrylic, polyfocal, refractive intraocular lens with a new optical design and geometry. This polyfocal IOL has three different zones (within the optic) with radially asymmetric design. Methods: We performed optical bench tests to calculate the optical characteristics of the sample. The optical performance and quality of IOLs based on ISO 11979-2 and 11979-9 requirements were analyzed with the NIMO TR0815 (Lambda-X). In addition, optical quality metrics were evaluated with the IOLA MFD device (Rotlex). Sphere, Add, modulation transfer function (MTF), the energy distribution between the modes and the MTF along the whole range from far to near were analyzed. Results: The power histogram showed that the tested IOL has the characteristics of a polyfocal IOL with a wide range of optical power between 20.5 and 24.5 diopters. Two distinct peaks were observed, indicating bifocal functionality. In the radial and axial power surface map, all three zones, stated by the company, could be detected. Larger apertures lead to a significant increase in MTF at the far peak, indicating better visual acuity for distant objects under low-light conditions. It was observed that in small aperture sizes, intermediate vision seems to be dominant. The energy distribution remained almost constant with increasing aperture size. Conclusions: This laboratory study was able to confirm the properties of the polyfocal lens stated by the company. Three optical zones could be identified. However, further optical bench tests should be performed to evaluate the new lens under tilted and decentered conditions. Clinical studies have to confirm that the presbyopia-correcting, polyfocal lens can achieve good clinical results with high patient satisfaction without disturbing side effects.

## 1. Introduction

The extraction of the cloudy crystalline lens and implantation of an intraocular lens (IOL) is routinely performed in age-related cataracts. This procedure has evolved continuously since the first IOL implantation by Sir Harold Ridley in November 1949 [1,2]. The surgical technique and the properties of the implant have improved steadily in the last 75 years [3,4]. This has led to a further increase in postoperative patient satisfaction. The improvements in regard to preoperative measurements of the eye (biometry) and more precise results due to new IOL power calculation formulas were decisive.

The implantation of a monofocal IOL results in an inability to accommodate for different distances. In more than 90% of cases (>30 million surgeries performed each year worldwide) so-called “standard monofocal lenses” are used [5]. However, the number of implanted premium lenses is growing as the desire for spectacle independence has grown in recent years. Cataract surgeries are often performed at an earlier stage of lens opacification due to the fact that the risk of surgical complications has decreased as a result of advances in technology [6,7,8]. New generations of IOL power calculation formulas have enormously increased the accuracy of the desired target refraction [9]. In addition to “classic” cataract surgery and the removal of the cloudy lens, refractive procedures known as “clear lens extractions” are becoming increasingly common to improve quality of life. Presbyopia-correcting or “premium” intraocular lenses have additional features such as astigmatism correction or multifocality of the optics to mimic the ability to see clearly at various distances by a simultaneous overlay of several images. Patients who desire spectacle independence usually have very high demands and expectations. The development of new optics in intraocular lenses together with improvements in biometry and better calculation formulas led to the success of those surgical procedures. By definition, multifocal IOLs incorporate multiple powers simultaneously, enabling different focal points at different distances. There are a variety of different presbyopia-correcting lenses with different optical principles on the market [10,11,12,13,14]. However, the large number of IOLs on the market and incomplete information from manufacturers on optical properties with advantages and disadvantages make it difficult for ophthalmologists/surgeons to select the best option in the individual case. In recent years, a large number of new IOLs with complex designs such as multifocal/polyfocal lenses, Extended Depth of Focus (EDOF) IOLs, and monofocal plus lenses with more and more subtypes based on their optical properties have been launched. It seems necessary for ophthalmologists to have a good overview of the available technologies in order to select the best option in each individual case. Therefore, the scientific, objective, and company-independent examination of these IOLs with laboratory studies and optical bench tests is crucial. However, these lab findings must always be seen in context with clinical studies and results from real-world scenarios. A recent study demonstrated a close correlation between laboratory-derived defocus curves and clinical outcomes [15].

The concept of trifocality has evolved considerably in recent times. Various designs have been presented. Multifocality in IOLs is achieved through refractive or diffractive optical approaches. Diffractive IOLs induce diffraction so that the waves exiting the lens will have constructive interference and several object planes are mapped to the same focus. Zonal refractive IOLs shape the waves exiting the lens from different annular regions where they converge. According to current knowledge, the leading approach of the last years appeared to be diffractive designs [16,17,18,19]. IOL models that try to utilize both principles are called hybrid diffractive-refractive lenses. Each lens model has its own characteristics that distinguish trifocality. This depends on the position of the intermediate and near points, which is determined by the distance between the refractive rings. In addition, the distribution of the light energy, which can be modulated by the height of the steps, determines overall performance. Pupil size also plays an important role in influencing the energy distribution (elimination of diffractive structures in mydriasis). In addition to correcting spherical corneal aberration, current lens designs also aim to influence chromatic aberration. In recent laboratory studies, trifocal IOLs have been assessed and their features have been identified. Optical quality was assessed using the modulation transfer function. Simulated defocus curves were derived from a non-linear formula [14]. The authors showed that these IOLs could be differentiated according to the position of the secondary foci position, light-energy distribution, and pupil-size-related behavior. It was stated that the add power of trifocal lenses and the exact location of intermediate and near focus were the main factors differentiating the tested IOLs. Some of the tested IOLs provided improved intermediate distance while others favored reading distance.

The aim of this laboratory study was to evaluate a newly launched polyfocal, refractive intraocular lens with a new optical design and geometry dimensions. The optical bench analysis was intended to illustrate the special features and properties (positive and negative) of the lens prior to the first clinical studies.

## 2. Material and Methods

### 2.1. Intraocular Lens

**Figure 1:**
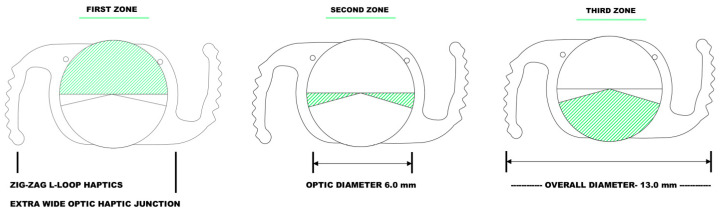
Schematic drawing of the newly launched hydrophobic, acrylic, polyfocal presbyopia-correcting intraocular lens (AutofocusPro, ModelNo VVB10SCA) showing the three optical zones and the “zig-zag L-loop” haptic design.

The Spirant AutofocusPro (Model No VVB10SCA by Lifeline Medical Devices Pvt.Ltd, Shendra, Aurangabad, India) intraocular lens with a power of 21.0 D was evaluated. It is a recently launched single-piece, hydrophobic, acrylic intraocular lens with an optical diameter of 6.0 mm and an overall diameter of 13.0 mm. According to the manufacturer’s specifications, the lens is made of a UV-absorbing material with a refractive index of 1.49 and an Abbe number of 43. The material of the IOL is produced by Contamac Ltd., Saffron Walden, United Kingdom. The lens has a 360° square edge design and is available in a power range of +3.0 D to +40.0 D. It has a theoretical A-constant of 118.5 and suggested A-constant (SRK/T 118.7, BARRETT’S LF 1.73). The oval-shaped optic aims to cover a larger area of the visual field, especially the temporal field of vision when the lens is placed horizontally. However, the recommended clear corneal incision size is 2.6 mm due to its special design. The lens is equipped with a novel, special haptic design (“zig-zag L-loop”) to provide (regarding the manufacturer’s claims) higher friction and solid rotational stability due to the high contact with the capsule. The anterior side of the optic is divided into 3 zones (Figure 1). The first zone (180–210°) for far distance, the second zone (5–15°) for intermediate distance, and the third zone (110–155°) for near distance. The diopter range for the second zone is (add +1.5 to 2.0 D). The posterior side of the lens has an aspheric design with a single zone.

### 2.2. Metrology Setup

Three identical lenses with the same power (21.0 D) were analyzed. We analyzed the samples on the optical bench using different systems and measuring devices to obtain objective and versatile results.

### 2.3. Optical Quality Metrics with NIMO TR0815

The NIMO TR0815, developed by Lambda-X (Nivelles, Belgium), is an advanced optical metrology instrument utilized for the precise characterization of intraocular lenses. This device employs state-of-the-art interferometry and wavefront analysis techniques to deliver high-resolution measurements of critical optical parameters. Specifically designed for refractive IOLs, the NIMO TR0815 provides comprehensive data on focal length, radius of curvature, and surface form error, among other parameters. Its integration of modulation transfer function (MTF) analysis further enhances its capability to assess optical performance and quality based on ISO 11979-2 and 11979-9 requirements.

The mechanism behind the NIMO TR0815 is based on the phase shifting principle (Interferometry), where a coherent light source (green) is split into two beams. One beam is directed towards the optical component under test, while the other serves as a reference. The two beams are then recombined, creating an interference pattern that is sensitive to minute deviations in the optical path length. This interference pattern, or interferogram, is captured by a high-resolution sensor and analyzed using sophisticated wavefront reconstruction algorithms. These algorithms enable the precise measurement of the wavefront aberrations introduced by the IOL, allowing for detailed characterization of its optical properties.

The calibration of the PMTF (power modulation transfer function) in optical metrology devices like the NIMO TR0815 and IOLA MFD involves ensuring that the devices provide accurate and reliable measurements of the modulation transfer function (MTF), which is critical for characterizing the optical performance of lenses or optical systems. The NIMO TR0815 and the IOLA MFD device use software calibration systems to compare the measured PMTF to known standards and apply corrections. Both devices typically follow standardized procedures for calibration, which may involve reference optical components, alignment checks, and software algorithms for data correction. Regular calibration is essential to maintain the accuracy and repeatability of PMTF measurements in optical metrology.

Metrology:

To calculate the optical characteristics of the samples under investigation, the following procedure was performed: first, the IOL sample was mounted carefully on the device’s sample holder, ensuring that it was securely positioned and free from any obstructions. Next, the optical axis of the IOL with the interferometer’s measurement beam was aligned. After calibration, the measurement process was initiated through the device’s user interface. The software captured the interferogram and applied wavefront reconstruction algorithms to analyze the optical characteristics of the sample, such as its power and spherical aberration.

### 2.4. Optical Quality Metrics Evaluated with the IOLA MFD Device

In evaluating optical performance, the following quantities were evaluated at any aperture: sphere, add, modulation transfer function (MTF) at each mode, and the energy distribution between the modes and the MTF along the whole range from far to near.

The IOLA MFD (Rotlex, Omer, Israel) is a model eye inspection system for IOLs. It consists of a point source on a moving rail and a pair of gratings that create a shearing interferometer. The software moves the point source to a position that is roughly the focal point of the IOL, thus yielding a pseudo-collimated beam after the cornea. The beam passes through the shearing gratings, thus creating a fringe pattern, which is processed by the software. The software utilizes the pupil correlation method for calculating the MTF and other proprietary mathematical methods for calculating the power map. Also, the unique optical design enables the scanning of MTF along 6–7 diopters in one shot, without moving the point source.

This allows for a detailed assessment of the optical quality and performance of the IOL samples. The Rotlex system’s sophisticated software facilitates comprehensive data analysis, visualization, and reporting. High-resolution wavefront reconstruction algorithms process the captured interferograms to quantify the optical properties of the IOLs, such as power, MTF, and aberrations.

## 3. Results

Optical Quality Assessment

The optical power map showed different results based on the analysis. Since this IOL has different zones (within the optic) with a radially asymmetric design, the radial power profile seemed to be not the most accurate way to analyze it. Therefore, it was necessary to evaluate in a specific mode, the so-called “multifocal mode” of the device.

The power histogram showed that the tested IOL has the characteristics of a polyfocal IOL with a wide range of optical power. In the radial and axial power surface map, it is possible to identify all three optical zones. However, in the spherical and cylindrical power surface map, it is only possible to identify different zones. Using a multi-aperture through-focus modulation transfer function (MTF) scan reveals that visual performance is affected by the aperture size.

**Figure 2:**
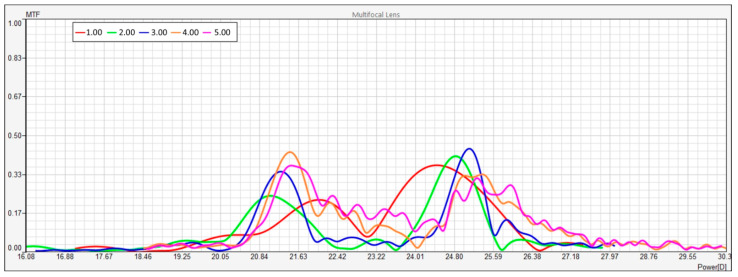
Multi-aperture MTF and power analysis for near and far focus. This figure presents the modulation transfer function (MTF) and optical power for both near and far focal points as a function of aperture size. The far power is represented by the green curve, while the near power is shown in orange. The red and blue lines correspond to the MTF at far and near focal points, respectively. The trends indicate how the optical performance and power vary with changes in aperture, highlighting the differences between near and far focus through various aperture sizes.

Figure 2 is a multi-aperture modulation transfer function (MTF) scan and through-focus MTF scan at several apertures. The red curve in the figure represents the modulation transfer function (MTF) at the smallest aperture, 1.00 mm. This curve shows moderate performance across the range of dioptric powers, with a peak at around 24.01 D. However, even at its highest point, the MTF value does not exceed 0.5, suggesting that the lens provides moderate contrast transfer at this aperture. The performance across other focal points is lower, with several minor peaks visible between 20 D and 30 D. Overall, at an aperture of 1.00 mm, the optical system demonstrates limited sharpness and less effective contrast transfer across various distances.

The green line, which corresponds to an aperture of 2.00 mm, exhibits higher MTF performance compared to the red line. The most pronounced peak occurs at 24.80 D, where the MTF value exceeds 0.5. This suggests that the lens provides sharper focus and better contrast transfer at this focal point. However, beyond this peak, the MTF decreases significantly, indicating that the lens’s sharp focus is confined to a relatively narrow range of defocus powers. At lower dioptric powers (between 16 D and 20 D), the performance is minimal. Thus, the lens at an aperture of 2.00 mm is most effective near 24.80 D, likely optimizing focus for intermediate or near vision distances.

The blue curve, representing an aperture of 3.00 mm, shows a complex pattern with several distinct peaks. The highest MTF values occur at around 20.84 D, 23.22 D, and 24.80 D, suggesting multiple focal points across the defocus range. The peak values approach 0.4, indicating reasonable contrast transfer, though not as high as with the green or pink lines. This aperture provides a balance between intermediate and far vision focal points but does not show the strongest performance in terms of sharpness. The contrast transfer quality drops off sharply beyond 25 D, suggesting limited effectiveness for more distant focus points.

The orange curve, corresponding to an aperture of 4.00 mm, shows a strong peak at around 21.63 D, where the MTF value approaches 0.5. This suggests that the lens is well-optimized for contrast transfer at this focal point. A secondary peak is also observed near 24.80 D, but the MTF values are lower compared to the primary peak. The orange line indicates better performance for intermediate distances but shows a more limited range of high MTF values, with sharp declines in performance both at higher and lower dioptric powers. Overall, the lens seems to provide reasonable focus at intermediate distances but lacks versatility across the entire defocus range.

The pink line, representing the largest aperture (5.00 mm), demonstrates the best overall performance, with multiple prominent peaks and the highest MTF values. The sharpest focus occurs at around 23.22 D, where the MTF value reaches 0.6, suggesting strong contrast transfer. This aperture performs well across a broader range of defocus powers compared to the others, with significant peaks between 22.42 D and 24.01 D. The lens’s optical performance at this aperture suggests that it is particularly effective at providing multifocal capabilities, delivering sharp focus over a broad range of powers.

In summary, the performance of the lens across different apertures varies, with larger apertures, such as 5.00 mm, showing better contrast transfer and a wider range of focal points. All apertures show peak performance between 20 D and 25 D, highlighting the distances for which the multifocal lens is optimized. Smaller apertures, such as 1.00 mm, provide less effective contrast transfer, while larger apertures demonstrate higher MTF values and better optical performance, especially under conditions with larger pupil sizes or brighter light. This variation across apertures reflects the multifocal nature of the lens and its capacity to provide sharp focus at multiple distances.

**Figure 3:**
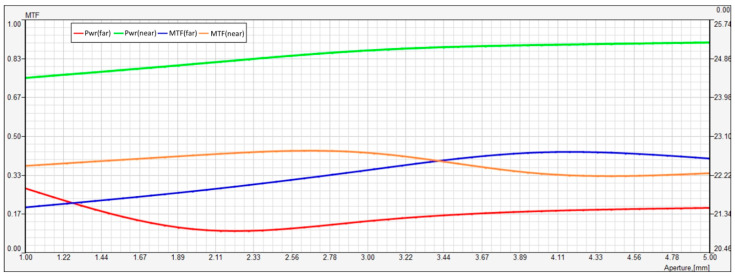
Relative intensity distribution for far and near vision across aperture sizes. This figure shows the relative intensity distribution (RNI) between far (red) and near (green) focal points as a function of aperture size. As aperture size increases, the near intensity decreases while the far intensity increases, crossing at approximately 3.4 mm. The results indicate a shift in energy distribution favoring distance vision at larger apertures.

Figure 3 represents a multi-aperture analysis showing the MTF and power (dioptric value) of a multifocal lens across varying aperture sizes from 1.00 mm to 5.00 mm. There are four curves presented: two power curves (far and near vision) and two MTF curves (far and near), corresponding to the lens’s performance at different focal points.

The green line shows the power for near vision across the aperture range. This curve remains relatively stable, increasing slightly from about 24.98 D at 1.00 mm to 25.74 D at 5.00 mm. The minimal variation indicates that the lens’s near vision power is highly consistent across different aperture sizes, suggesting stable refractive performance for near objects.

The red line corresponds to the power for far vision. The power value for far vision starts at about 21.34 D at the smallest aperture (1.00 mm) and steadily increases as the aperture widens, reaching around 21.74 D at 5.00 mm. This slight increase in power suggests a small shift in far vision focal power as the aperture enlarges, which could be due to changes in the lens’s optical properties when more of the lens surface is exposed.

The orange line represents the MTF for near vision. The MTF remains relatively stable across the aperture sizes, starting around 0.34 at 1.00 mm and showing a slight increase, reaching about 0.37 at 5.00 mm. This indicates that the lens’s ability to transfer contrast for near vision remains consistent and improves slightly as the aperture size increases. However, it seems that it does not change drastically across the range.

The blue line corresponds to the MTF for far vision. At 1.00 mm, the MTF for far vision is around 0.17, and it steadily increases as the aperture widens, reaching approximately 0.34 at 5.00 mm. This suggests that the lens performs better for far vision at larger apertures. It seems that as more light enters through a larger aperture, the lens is able to transfer more contrast, improving vision quality at far distances.

In summary, this figure demonstrates that both near and far vision power remains relatively stable across all aperture sizes, with slight increases in both as the aperture widens. The MTF for far vision shows significant improvement with larger apertures, while the MTF for near vision remains relatively consistent with only a minor improvement. This behavior highlights the lens’s ability to maintain good optical performance across a range of aperture sizes, with improved contrast transfer for far vision at larger apertures.

**Figure 4:**
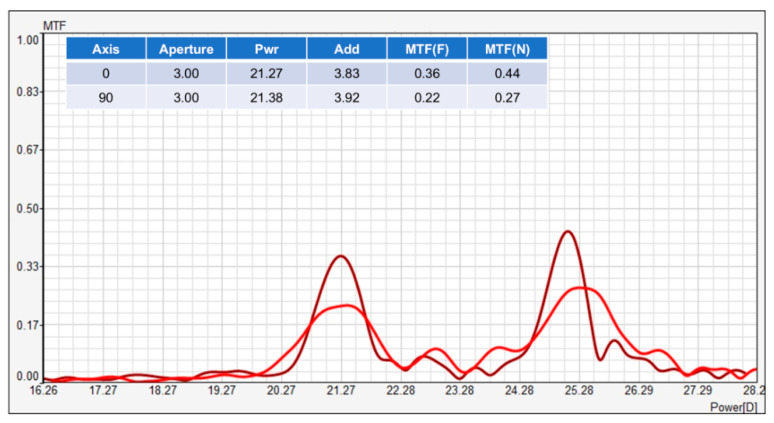
Through-focus modulation transfer function of a multifocal lens. This figure illustrates the through-focus modulation transfer function (MTF) for a bifocal multifocal lens along two perpendicular meridians, at 0° (dark red line) and 90° (light red line). Peaks in the MTF curves correspond to the near and far focal points. The optical power ranges between 16.26 D to 28.2 D, with higher MTF values indicating better resolution at those specific focal points.

Figure 4 presents the Through Focus Resolution (TFR) of a bifocal multifocal lens, displaying modulation transfer function (MTF) values along two perpendicular meridians (axis 0° and 90°) with an aperture of 3.00 mm. The MTF data are plotted against dioptric power, showing the lens’s optical performance at various focal points.

Two sets of MTF curves are shown for axes 0° and 90°, representing different orientations of the lens. Each meridian has corresponding MTF peaks at different dioptric powers, indicating focal points where the lens provides higher contrast transfer.

The darker red curve represents the MTF along the 0° axis. For this meridian, the MTF has a prominent peak at approximately 21.27 D, corresponding to far vision. Another noticeable peak appears at around 25.28 D, which corresponds to near vision. These peaks demonstrate the bifocal design’s ability to focus on two distinct distances—far and near—as intended by the lens design.

The lighter red curve corresponds to the 90° axis. This meridian has a similar overall pattern but shows slightly reduced MTF values at both far and near vision peaks. For the far focal point at 21.38 D, the MTF is lower than for the 0° axis, and the near vision peak around 25.28 D is also somewhat lower. This indicates some asymmetry in the optical performance between the two meridians, which could be attributed to differences in the design or possible optical aberrations.

The table at the top of Figure 4 summarizes the key performance metrics for the two meridians. The power (Pwr) values for far vision are 21.27 D and 21.38 D for the 0° and 90° meridians, respectively. The add power (Add), which indicates the additional power for near vision, is approximately 3.83 D for the 0° meridian and 3.92 D for the 90° meridian. The MTF values for far and near vision show higher contrast transfer for the 0° meridian, with MTF(F) = 0.36 and MTF(N) = 0.44. For the 90° meridian, these values are lower, with MTF(F) = 0.22 and MTF(N) = 0.27.

In summary, Figure 4 illustrates the TFR of a bifocal multifocal lens, showing that the lens performs better along the 0° meridian than the 90° meridian in terms of MTF for both far and near vision. The peaks in the MTF curves at around 21.27 D and 25.28 D for both meridians clearly correspond to the far and near focal points, as expected for a bifocal lens.

**Figure 5:**
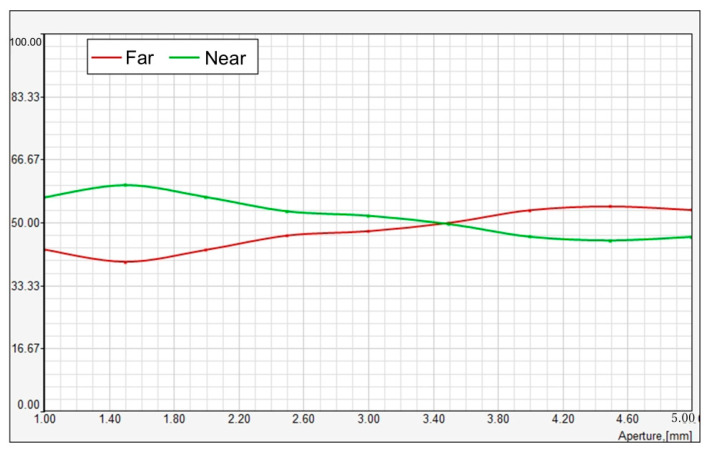
Power and Energy Distribution for Near and Far Focus with Aperture Size. This figure shows the distribution of optical energy between the near (green) and far (red) focal points as aperture size changes. The near energy distribution de-creases with increasing aperture, while far energy increases, demonstrating how optical performance for both distances is affected by aperture size.

Figure 5 represents the relative energy distribution (RNI) of light between far and near focal points in a bifocal lens system as a function of aperture size (measured in mm). Two curves are plotted: red for far vision and green for near vision.

The green curve indicates the relative energy distribution for near vision, which starts at a higher value when the aperture is small (around 1.0 mm), signifying that a greater proportion of light energy is directed towards the near focus for smaller apertures. As the aperture size increases, the energy distribution gradually decreases, reaching a minimum at around 3.40 mm where it crosses the red curve.

The red curve represents the relative energy distribution for far vision, which starts at a lower value compared to near vision for small apertures. As the aperture increases, the energy distributed to far vision grows steadily and surpasses the near energy distribution at approximately 3.40 mm. For apertures larger than 3.40 mm, the far vision energy continues to rise, stabilizing beyond 4.0 mm.

The crossing point of the two curves at around 3.40 mm indicates an equilibrium where the energy distribution between far and near is approximately equal. For smaller apertures, more energy is allocated to near vision, while for larger apertures, more energy is directed towards far vision.

## 4. Discussion

With 2 mm and 3 mm apertures, the MTF scan showed improved bifocal performance characterized by pronounced double peaks. This suggests that these apertures are even more effective in providing clear vision at multiple focal distances. Under conditions of dim illumination, which causes the pupil to dilate to diameters of 4 mm or 5 mm, there is a notable enhancement in MTF across the entire range of vision, spanning from distant to near (intermediate) vision. This dilation (large aperture) leads to a significant increase in MTF at the far peak, indicating better visual acuity for distant objects under low light conditions. The characteristic behavior of the tested polyfocal lens aligns with the optical design of multifocal lenses, where energy distribution varies with aperture size, affecting the lens’s focal priorities.

The MTF peaks for multi-aperture mode exhibited a gradual increase with the aperture size, indicating that the quality of vision remains stable even as illumination decreases. This characteristic could be advantageous, particularly because it is accompanied by an improvement in intermediate vision. However, clinical tests must confirm this.

Trifocal IOLs are an emerging technology that offer intermediate vision in addition to distance and near. It is well known that trifocal IOL models use different technologies and designs. The aim is always to improve intermediate and near vision without deteriorating distance vision systematically. Avoiding and reducing disruptive side effects such as halo, glare, starburst, and reduced contrast should be the overriding principle. Various designs have been introduced in recent years, leading to different results with advantages and disadvantages. The main distinction between the different optical approaches to achieve multifocality is that the out-of-focus light in the diffractive IOLs tends to be spread out uniformly over a larger area and is thereby less noticeable. The out-of-focus light in zonal refractive multifocal lenses is concentrated into rings around the objects and cannot be easily suppressed. As a result, diffractive IOLs have replaced zonal refractive multifocal lenses in many cases.

It is well known from the literature that side effects such as halo, glare, starburst and blurred vision with reduced contrast sensitivity have to be evaluated and compared. In the past, many clinical studies mainly reported visual acuity and did not consider side effects. In other studies, the number of cases was relatively small. Therefore, optical bench studies are required to obtain objective data on new IOL designs. This is particularly important for IOLs that are newly launched. Recently, a study investigated and compared the optical performance of five, new trifocal intraocular lenses following the ISO 11979-2 standards, analyzing the impact of tilt and decentration [20]. The five tested IOLs had different optical principles. In-vitro optical quality analysis was performed with the Lambda PMTF system and the VisIOLA system (Rotlex) and measurements were performed on-axis, with 5° of IOL tilt and with 0.5 mm of IOL decentration using 543 nm monochromatic light. The authors summarized that there were differences in the optical performance according to the aperture of the five tested IOLs and that tilt and decentration significantly affected the performance of all IOLs at the intermediate vision range. Decentration and tilt lead to a significant reduction in the modulation transfer function (MTF) and, depending on the optical design, to less or more interference in the near/intermediate or far range. Another study determined the impact of spherical and aspheric IOL tilt and decentration on optical quality after cataract surgery [21]. Tilt and decentration of the IOLs were measured using Scheimpflug photography and the effect of tilt and decentration on higher-order aberrations and optical quality was assessed using multiple regression analysis. The authors found a mean optic tilt of 2.85 degrees ± 1.36 and a mean decentration of 0.27 mm ± 0.16. It is most important for IOLs to function within the commonly occurring range of tilt and decentration. According to simulations and considering aspects such as corneal aberrations, pupil function, and other factors influencing visual performance, IOL decentrations of >0.5 mm can lead to significant visual degradation [22]. Another paper reported that optical quality was significantly reduced at all distances for diffractive bifocal and trifocal IOLs if the IOL decentration exceeded 0.75 mm, with intermediate focus showing the least reduction [23]. It was proven by optical bench tests that monofocal, aberration-correcting IOLs perform best when perfectly centered [24]. The optical performance of monofocal, aberration-correcting IOLs was markedly downgraded by misalignment. Another experiment showed that tilt and decentration also had a major impact on non-diffractive extended range of vision intraocular lenses with larger aperture sizes [25]. Experiments have also shown that the design and geometry of the lens, including the optic-haptic junction and the shape of the haptics may affect the behavior of the IOL in different radial zones [26]. This fact seems to be important as it affects the contact of the lens to the capsule in real-world scenarios. This is apparently crucial for stable positioning of the IOL and a lower probability of tilt/decentration. However, laboratory experiments cannot represent real life, which is why multicenter clinical studies are particularly important. Overall, it is clear that the technology of presbyopia-correcting IOLs has developed enormously in recent years. It is an advantage for the user/ophthalmologist and the recipient/patient that there are so many different models of IOLs with different optical principles on the market. It gives the opportunity to choose the best option for the individual case. However, an appropriate background knowledge of the working principle and the differences between designs is required. Moreover, it has been shown that side effects such as halos and glare still occur. This is also the reason why selecting different lens types for two eyes as a “mix-and-match approach” exists to exploit advantages and reduce disadvantages. Recently, a study confirmed that combining an EDOF IOL (dominant eye) and a typical multifocal lens (non-dominant eye) was well tolerated by subjects and provided some potential benefits relative to bilateral implantation of either lens [27]. Visual acuity was very satisfying but halos were still the disturbance reported most frequently and reported as most bothersome, with some difficulty driving at night as the most common visual function issue. Similar studies using this “mix-and-match approach” reported less severe visual disturbances [28,29,30,31]. When new IOLs are launched, independent laboratory experiments are important to demonstrate the properties of the IOL. However, clinical evaluations that follow are just as important for making statements about quality, properties, and indications. Conclusive recommendations on the use of new lenses can only be made after considering all of the results.

Limitations of the study

Our optical bench study is only an initial analysis of the optics of this newly launched polyfocal lens. Further optical bench tests have to be performed to show optical performance under tilted/decentered conditions. No statements can be made regarding clinical performance (neither positive properties nor negative side effects).

## 5. Conclusions

Our laboratory study was able to confirm the features and properties of a polyfocal lens with a wider range of optical power as stated by the manufacturer. In the radial and axial power surface map, it was possible to identify all three optical zones. The data showed that in small aperture sizes (1 to 3 mm), intermediate vision seems to be dominant. When it comes to larger aperture sizes, far vision seems more dominant and the energy distribution remained almost constant. However, there remain some limitations in the most accurate measurement of such polyfocal lenses. Furthermore, our results cannot be directly transferred to clinical practice as these are purely experimental data. Clinical studies are recommended with long-term observation to evaluate = overall performance and patient satisfaction. Moreover, this study demonstrates the important context of aperture size/pupil size and visual performance. The results highlight how lighting conditions can impact the quality of vision through changes in the MTF.

## Data Availability

The original contributions presented in this study are included in the article. Further inquiries can be directed to the corresponding author.
